# Continuous Suprascapular Catheter and Axillary Nerve Block for Analgesia for Reverse Total Shoulder Arthroplasty: A Case Report

**DOI:** 10.7759/cureus.49670

**Published:** 2023-11-29

**Authors:** Ammar Toubasi, Dylan S Irvine, Karim Jandali, Daniel Sweeney, Sebastian M Monasterio

**Affiliations:** 1 Anesthesiology and Perioperative Medicine, Augusta University Medical College of Georgia, Augusta, USA; 2 College of Medicine, Nova Southeastern University Dr. Kiran C. Patel College of Osteopathic Medicine, Davie, USA

**Keywords:** ultrasound-guided axillary nerve block, regional anesthesiology, suprascapular nerve block, reverse total shoulder arthroplasty, regional nerve block

## Abstract

Reverse total shoulder arthroplasty (RTSA) is a widely employed surgical intervention for managing advanced shoulder arthritis. Postoperatively, patients frequently experience intense pain, particularly within the first 48 hours. Effective pain management through regional analgesia not only facilitates a quicker hospital discharge but also minimizes the reliance on opioids. One such regional analgesic approach is the combined suprascapular and axillary nerve block, which targets the glenohumeral joint, rotator cuff muscles, and the shoulder's lateral region for effective pain alleviation. Previous research indicates that this dual nerve block method offers sustained pain relief while circumventing the respiratory complications commonly associated with interscalene brachial plexus blocks, which may inadvertently block the phrenic nerve and affect respiration. We report the case of a 75-year-old female, diagnosed with severe chronic obstructive pulmonary disease (COPD) and bronchiectasis on multiple inhalers, who presented for RTSA. The patient had a strong desire to avoid opioids for pain control due to adverse side effects. Through a suprascapular nerve catheter and axillary nerve single shot, regional analgesia was administered, which minimized the risk of respiratory complications due to potential phrenic nerve involvement from an interscalene approach. There were no opioids taken in the postoperative period after discharge, and the patient only received oral acetaminophen. The patient experienced a successful recovery without any respiratory complications and was extremely satisfied with her management.

## Introduction

Reverse total shoulder arthroplasty (RTSA) represents a widely adopted surgical intervention for addressing advanced shoulder arthritis. This procedure is often associated with intense postoperative pain, especially within the first 48 hours, necessitating effective pain management strategies. Regional analgesia has emerged as a crucial element in this context, not only facilitating early hospital discharge but also reducing the reliance on opioids for pain relief [1]. One promising approach to regional analgesia is the combined suprascapular and axillary nerve blocks. This technique targets specific anatomical structures, including the glenohumeral joint, rotator cuff muscles, and the shoulder's lateral region, to provide effective pain alleviation [2]. Prior research supports the use of this dual nerve block method, as it offers sustained pain relief while mitigating the respiratory complications often associated with the phrenic nerve block in interscalene brachial plexus block [3].

## Case presentation

We present the case of a 75-year-old female patient scheduled for reverse total shoulder arthroplasty (RTSA). Her past medical history included severe chronic obstructive pulmonary disease (COPD) and bronchiectasis, for which she used albuterol as needed, moderate obstructive sleep apnea (OSA), managed with continuous positive airway pressure (CPAP), as well as secondary hyperparathyroidism, osteoporosis, gastroesophageal reflux disease (GERD), and cardiovascular conditions such as coronary artery disease (CAD), hypertension (HTN), and atrial fibrillation (AF). Past surgical history included a maze procedure for AF and pacemaker implantation for sick sinus syndrome (SSS) and tachy-brady syndrome. The patient was taking flecainide and apixaban, with known allergies to ibuprofen, oxycodone, and tramadol.

**Figure 1:**
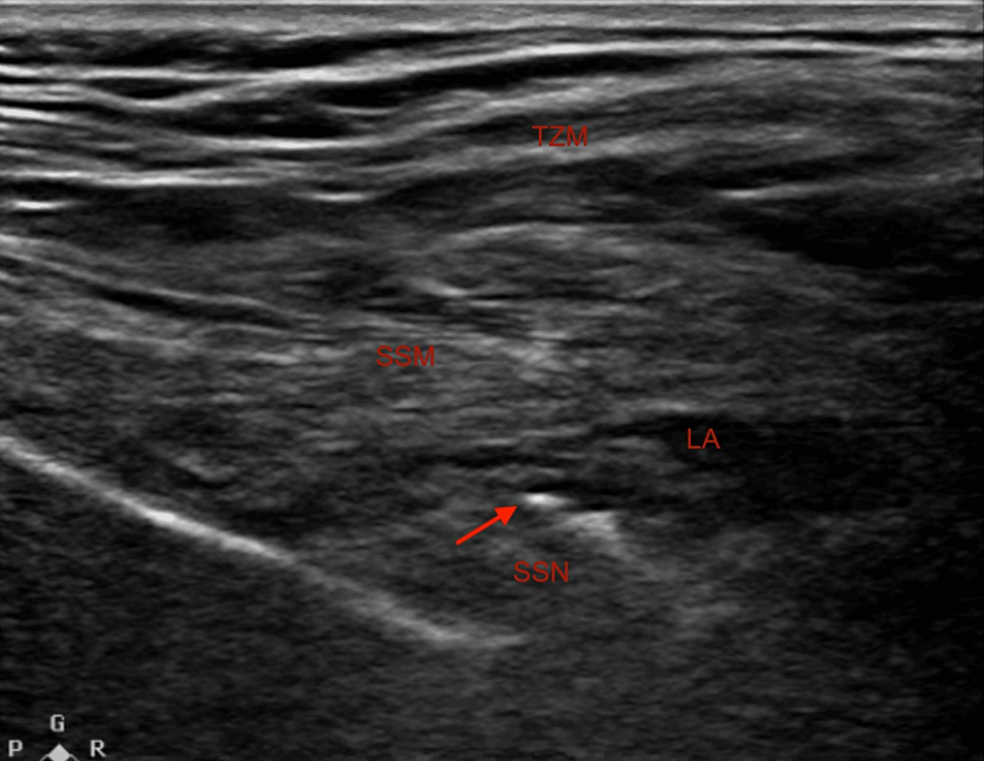
Ultrasound-guided placement for the suprascapular nerve block. TZM: trapezius muscle; SSM: supraspinatus muscle; LA: local anesthetic; SSN: suprascapular nerve; Arrow: needle tip

**Figure 2:**
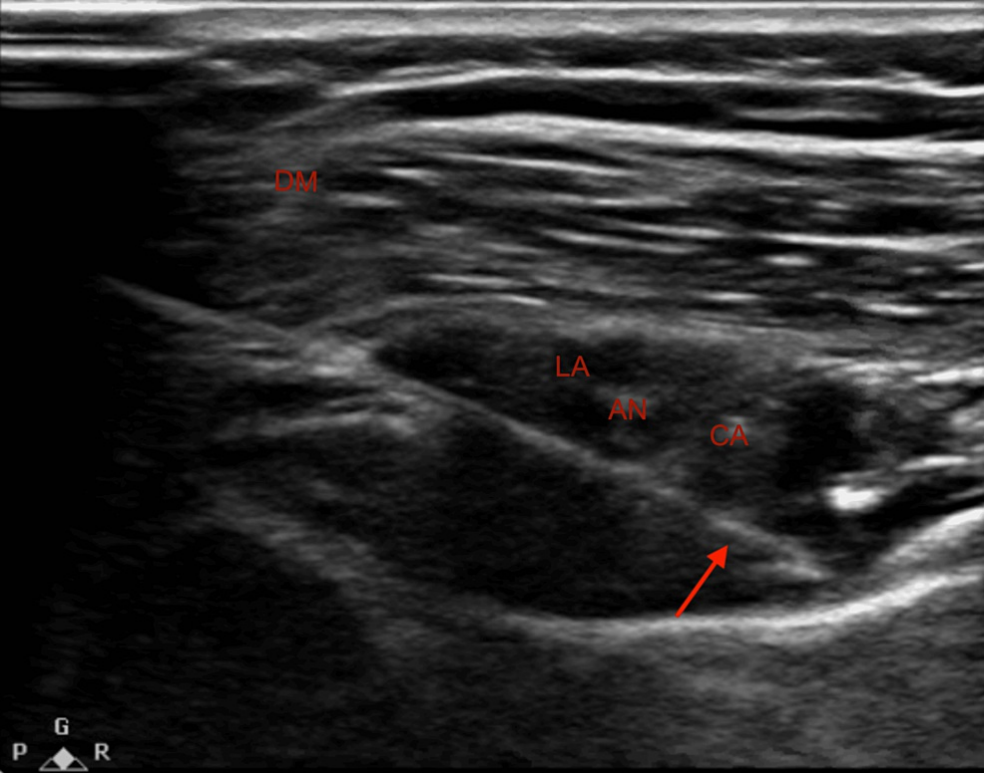
Ultrasound-guided needle placement for the axillary nerve block. DM: deltoid muscle, AN: axillary nerve, CA: circumflex artery, LA: local anesthetic, Arrow: needle tip

In the preoperative period, both blocks were performed. The suprascapular nerve block was performed using the posterior technique by placing the transducer above the spine of the patient's scapula to visualize the trapezius and the supraspinatus muscle, as shown in Figure 1. The needle was advanced with an in-plane approach from medial to lateral through the muscles until the tip of the needle was below the supraspinatus fascia and adjacent to the suprascapular artery and nerve. Once confirmed that it was not intravascular by negative aspiration, 10 cubic centimeters (cc) of 0.5% ropivacaine were administered, followed by the insertion of a catheter into the suprascapular space [4]. Careful attention was given to avoiding catheter placement within the surgical field. Additionally, a single-shot axillary nerve block was done, as shown in Figure 2. By placing the transducer on the posterior aspect of the shoulder, the neck and shaft of the humerus, the deltoid muscle, teres minor muscle, and the circumflex artery were identified, parallel to the longitudinal axis of the shaft of the humerus. Using the in-plane technique, the needle was advanced until its tip entered the interfascial space between the deltoid muscle and the teres minor muscle, and then 15 cc of 0.5% ropivacaine was administered [5].

For induction of general anesthesia, the patient received 200 micrograms (mcg) of fentanyl, as well as the appropriate induction medications utilized for a standard induction, with no need for additional analgesics. During recovery, a single dose of 0.5 milligrams (mg) of hydromorphone was administered for postoperative pain management. Postoperatively in the post-anesthesia care unit, a continuous infusion of 0.2% ropivacaine at a rate of 10 cc per hour (cc/hr) was initiated for analgesia.

The patient was discharged on the first postoperative day with an ON-Q pump (I-Flow Corp, Lake Forest, California, USA) preloaded with 600 cc at a rate of 10 cc/hr, providing 60 hours of continuous infusion for effective pain control. On the second postoperative day, the patient reported being pain-free for 48 hours without experiencing any complications. She used acetaminophen 650 mg one to two times a day for supplementary pain relief. By the 14th postoperative day, the patient expressed her satisfaction with the level of pain relief achieved, and notably, no opioids were required.

## Discussion

Arthroscopic shoulder surgery frequently results in severe intraoperative and postoperative pain, affecting nearly 45% of patients [6]. This pain often proves substantial enough to disrupt the early stages of recovery and rehabilitation [6]. Interscalene brachial plexus block (IBPB) has been commonly used for pain relief in RTSA. However, complications associated with IBPB like high spinal, phrenic nerve palsy, and dyspnea can be particularly concerning for patients with moderate/severe COPD, such as our patient [7]. The suprascapular and axillary nerve blocks provide effective anesthesia for arthroscopic shoulder procedures while minimizing respiratory risks such as dyspnea or respiratory compromise associated with more proximal brachial plexus blocks. 

In our patient with severe COPD and bronchiectasis, who was on multiple inhalers, traditional anesthesia techniques like the interscalene nerve block were considered but decided against due to potential respiratory complications such as dyspnea (most commonly) and pneumothorax. The combined use of suprascapular and axillary nerve blocks emerged as a safer alternative, providing effective pain management without compromising respiratory function. This technique's ability to offer targeted anesthesia while preserving both motor function and respiratory integrity made it particularly suitable for our patient, highlighting its potential benefits for similar cases, especially when used in conjunction with peri-articular infiltration (PAI) anesthesia [8]. Other studies such as Neuts et al. and Dhir et al. have demonstrated that the combined suprascapular and axillary nerve blocks are advantageous over other approaches due to a lower incidence of complications such as dyspnea and discomfort [3,9]. Our patient's case underscores the importance of individualized anesthesia planning, especially in those with significant respiratory comorbidities such as moderate or severe COPD and bronchiectasis. The findings from Neuts et al., Dhir et al., and our own observations emphasize the need for healthcare providers to be vigilant in assessing patient-specific risk factors [3,9]. In the future, a more standardized approach to anesthesia selection, based on a comprehensive evaluation of patient history, comorbidities, and potential drug interactions, could further optimize patient outcomes.

Providers should be particularly attentive to patients with multiple comorbidities or those with pre-existing respiratory conditions, ensuring that anesthesia techniques align with the patient's unique needs and minimize potential complications. In our case, the patient fit into the algorithm where the combined nerve blocks were the optimal choice due to the aforementioned respiratory concerns. Reflecting on literature reviews and our patient's outcome, we believe the patient presented in this case was managed appropriately. Further studies and experience using the approach documented in this case could influence the management of similar patients in the future. Providers should be encouraged to adopt a more algorithmic approach, ensuring that each patient receives care tailored to their specific needs and conditions. The goals of anesthetic management in these scenarios extend beyond just pain control; they encompass ensuring respiratory stability and optimizing overall surgical outcomes. It is imperative for anesthesia providers to have a comprehensive understanding of the patient's medical history, the potential risks associated with traditional anesthesia techniques, and the benefits of alternative approaches like the combined suprascapular and axillary nerve blocks. Strategies such as thorough pre-operative assessments, interdisciplinary discussions, and continuous intraoperative monitoring can significantly optimize outcomes. This case underscores the pivotal role anesthesia plays in the holistic management of patients, emphasizing the need for individualized care and a deep understanding of the broader clinical picture. The goals of anesthetic management in these scenarios extend beyond just pain control; they encompass ensuring respiratory stability, minimizing potential drug interactions, especially with inhalers, and optimizing overall surgical outcomes.

Rapid identification and intervention for potential complications are paramount in enhancing patient outcomes and averting severe morbidity and mortality. When devising the anesthesia care plan for regional blocks and approaches to postoperative analgesia, providers should weigh the patient's respiratory status, potential drug interactions, especially with multiple inhalers, and the risks associated with traditional anesthesia techniques. Emphasis should also be placed on the benefits of alternative approaches, such as the combined suprascapular and axillary nerve blocks. Future research should delve into the long-term outcomes and potential complications of these alternative anesthesia techniques, providing a more comprehensive understanding and guiding best practices for similar patient profiles.

## Conclusions

In consideration of the reduced risk of side effects and complications, the combined suprascapular and axillary nerve blocks were selected as the analgesic approach for our patient, who had multiple respiratory and cardiovascular comorbidities. This is not to imply that the interscalene nerve block is inferior; rather, the combined approach may be particularly beneficial for high-risk populations, especially those with respiratory complications. In our case, we wanted to emphasize the importance of tailoring analgesic strategies to high-risk patients. Rather than solely comparing the effectiveness of this nerve block to others, our focus is on the decision-making process that led to choosing this specific block. We considered the patient's characteristics, opting for a block with a lower likelihood of complications.
